# Peptide-Based Optical uPAR Imaging for Surgery: *In Vivo* Testing of ICG-Glu-Glu-AE105

**DOI:** 10.1371/journal.pone.0147428

**Published:** 2016-02-01

**Authors:** Karina Juhl, Anders Christensen, Morten Persson, Michael Ploug, Andreas Kjaer

**Affiliations:** 1 Department of Clinical Physiology, Nuclear Medicine & PET and Cluster for Molecular Imaging, Rigshospitalet and University of Copenhagen, Copenhagen, Denmark; 2 Department of Otolaryngology, Head & Neck Surgery and Audiology, Rigshospitalet, Copenhagen, Denmark; 3 Finsen Laboratory, Rigshospitalet, Copenhagen, Denmark; 4 Biotech Research and Innovation Centre (BRIC), Copenhagen University, Copenhagen, Denmark; University of Freiburg, GERMANY

## Abstract

Near infrared intra-operative optical imaging is an emerging technique with clear implications for improved cancer surgery by enabling a more distinct delineation of the tumor margins during resection. This modality has the potential to increase the number of patients having a curative radical tumor resection. In the present study, a new uPAR-targeted fluorescent probe was developed and the *in vivo* applicability was evaluated in a human xenograft mouse model. Most human carcinomas express high level of uPAR in the tumor-stromal interface of invasive lesions and uPAR is therefore considered an ideal target for intra-operative imaging. Conjugation of the flourophor indocyanine green (ICG) to the uPAR agonist (AE105) provides an optical imaging ligand with sufficiently high receptor affinity to allow for a specific receptor targeting *in vivo*. For *in vivo* testing, human glioblastoma xenograft mice were subjected to optical imaging after i.v. injection of ICG-AE105, which provided an optimal contrast in the time window 6–24 h post injection. Specificity of the uPAR-targeting probe ICG-AE105 was demonstrated *in vivo* by 1) no uptake of unconjugated ICG after 15 hours, 2) inhibition of ICG-AE105 tumor uptake by a bolus injection of the natural uPAR ligand pro-uPA, and finally 3) the histological colocalization of ICG-AE105 fluorescence and immunohistochemical detected human uPAR on resected tumor slides. Taken together, our data supports the potential use of this probe for intra-operative optical guidance in cancer surgery to ensure complete removal of tumors while preserving adjacent, healthy tissue.

## Introduction

Development of improved methods for cancer resection has in many years been relatively stagnant. The current surgical principle is to differentiate healthy from diseased tissue under white light illumination by direct visual inspection and palpation. This can in many cases be difficult due to an irregularly shaped invasive front and microscopic tumor deposits. In cancer treatment the best prognosis is associated with complete removal of the cancerous tissue [1–4]. At present, the gold standard for assessment of optimal resection with tumor-free margins, is postoperative histological examination of the resected tumor specimen and tumor bed [5]. Intraoperative assessment of tumor margins by frozen samples is time consuming and less accurate compared to postoperative histopathological examination [6]. Incomplete tumor resections remains a major challenge for numerous solid cancers [7,8] and emphasise the need for a better and improved techniques for tumor resection.

Intraoperative optical imaging employing targeted near infrared (NIR) spectral probes is a novel technique allowing surgeons to differentiate tumor from non-cancerous tissue [9,10]. NIR fluorophors (NIRF) are advantageous for intraoperative imaging compared to other widely used fluorophors with lower excitation wavelength maxima, due to the higher penetration depth of ½-1 centimetre seen with NIRF [11]. Moreover, tissue auto-fluorescence is limited in the NIR range (650–900nm) and therefore increases the tumour to background ratio (TBR) to a level needed for intraoperative imaging. These properties make NIRF useful in optical-guided surgery. However, light emission in this wavelength range is invisible for the human eye and a camera system is therefore needed to visualize the distribution of the optical probe in the surgical field.

Urokinase-type plasminogen activator receptor (uPAR) is over-expressed in many solid cancers, including glioblastomas, breast, colorectal and prostate cancer [12–14]. High expression levels of uPAR are generally associated with poor prognosis and metastatic dissemination and the receptor is often located in excess at the invasive front of the tumor and in the adjacent stroma [4]. This expression pattern makes uPAR an ideal target for intraoperative optical imaging. Development of a high-affinity 9-mer peptide (AE105) targeting human uPAR [15], has been instrumental for our design of PET-probes for the non-invasive detection of uPAR expressing cells and their subsequent eradication by uPAR targeted radiotherapy [16–19]. In the present study, we conjugated AE105 with indocyanine green (ICG) for the development of an uPAR-targeted optical probe. ICG as fluorophore was approved for clinical use more than 50 years ago and has been used e.g. for retinal angiography and hepatic clearance [20].

The aim of the present study was therefore to characterize a new variant of AE105 suitable for optical imaging (ICG-Glu-Glu-AE105) both *in vitro* and *in vivo* for its potential use in fluorescent-guided cancer surgery.

## Materials and Methods

### Chemistry

**Fig 1:**
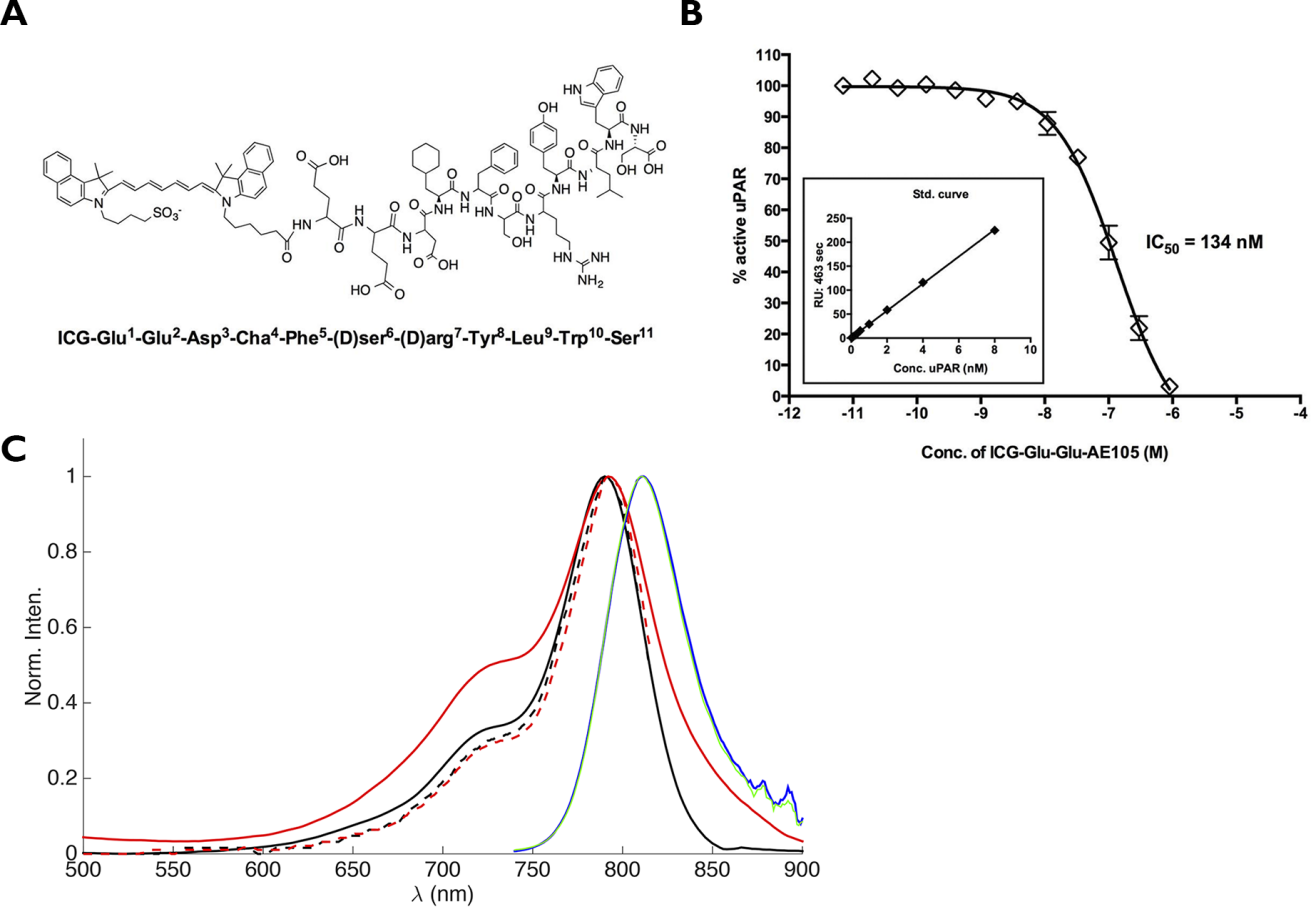
Structure and binding affinity for ICG-Glu-Glu-AE105. (A) The chemical structure of ICG-Glu-Glu-AE105 (B) Assessing uPAR binding properties of ICG-Glu-Glu-AE105 by an indirect solution competition for uPA binding by surface plasmon resonance yielding an IC_50_ value of 134 nM. (C) Absorption spectra of ICG (black, full line) and ICG-Glu-Glu-AE105 (red, full line) measured in HBC solution. Ecitation spectra of ICG (black, broken line) and ICG-Glu-Glu-AE105 (red, broken line) measured in HBC solution. Fluorescence spectra of ICG (blue) and ICG-Glu-Glu-AE105 (green) measured in HBC solution. The noise observed between 860–900 nm in the fluorescence spectra are due to poor detector correction of the instrument in this region.

The peptide AE105 [21] including an N-terminal extension by two glutamic acid residues was conjugated via its α-aminogroup to ICG (4-(2-((1E,3E,5E,7Z)-7-(3(5-carboxypentyl)-1,1-dimethyl-1H-benzo[e]indol-2(3H)-ydlidene)hepta-1,3,5-trienyl)-1,1dimethyl-1H-benzo[e]indolium-3-yl)butane-1-sulfonate) (ICG-Glu-Glu-AE105, Fig 1A) was purchased from ABX (Radeberg, Germany). The purity of the final product was more than 99%. For *in vivo* injection ICG-Glu-Glu-AE105 was dissolved in (2-hydroxypropyl)-β-cyclodextrin with 2% DSMO. Recombinant human pro-uPA was produced and purified as described previously [22].

### Cell lines and animal set-up

The human glioblastoma cell line U87MG was purchased from the American Type Culture Collection (Manassas, VA, USA) and culture media was obtained from Invitrogen (Paisley, UK). U87MG was cultured in Dulbecco’s modified eagle medium added 10% FBS and 1% PenStrep. When cells reached 70–80% confluence they were harvested.

All animal experiments were performed under a protocol approved by the Animal Research Committee of the Danish Ministry of Justice (2012-15-2934-00064). 5 ×10^6^ U87MG cells were suspended in 200 ul PBS and inoculated in both flanks of 6 weeks old nude NMRI female mice (24–28 g; Taconic Europe, Lille Skensved, Denmark). All mice were housed in groups with 12 h light/dark cycle and food and water was available ad libitum. When the tumours reached approx. 500 mm^3^ in size the mice were enrolled in the optical imaging protocol.

### Measurement of the physiochemical properties

The UV-vis absorption spectra were recorded on a Perkin-Elmer Lambda 1050 UV/vis/NIR spectrometer double beam spectrometer using the HBC solvent as baseline. The spectra were recorded in 1 cm path length cuvettes with ICG and ICG-AE105 present in the 10–50 μM range. Excitation and emission spectra and lifetimes were measured using FluoroTime 300 (PicoQuant, Berlin, Germany) system. The spectra were measured through excitation at 725 nm using a Xe-lamp excitation source. The excitation and emission measurements were measured in 1 cm cuvettes at 90° with respect to the excitation light. In the fluorescence measurements the absorbance of the samples were below 0.1 at the maximum of the lowest energy absorption band to avoid inner filter effects. After each fluorescence measurement the absorption spectra were recorded in order to verify that no photobleaching of the sample had occurred during the fluorescence measurement. All excitation and fluorescence spectra were corrected for the wavelength-dependent sensitivity of the detection from 500–900 nm.

### Surface plasmin resonance studies

The IC_50_-value for ICG-Glu-GLU-AE105 as competitive inhibitor of the uPA•uPAR interaction was measured by surface plasmon resonance in a Biacore3000 assay platform as previously published [23]. Subsequent fitting to the binding isotherms by non-linear regression provided the IC_50_-value.

### Plasma stability of ICG-Glu-Glu-AE105

ICG-AE105 (5 μg in DMSO) was added to 150 μl mouse plasma and the solution was incubated at 37°C for either 0, 1, 5 or 30 minutes. After incubation, acetonitrile (200 μl) was added followed by centrifugation at 3500 rpm for 4 minutes. The supernatant was collected, diluted and passed through a 0.45 μm filter. The filtered solution was analyzed on a Dionex UltiMate 300 HPLC using an Onyx Monolithic column (Phenomenex, 50x4.6 mm) and with a flow of 1.5 ml/min. The HPLC mobile phase was 0.1% TFA in H_2_O:MeCN 90:10 (A) and 0.1% TFA in H_2_O:MeCN 10:90 (B) and the gradient program: 0–2 min 10% B, 2–20 min 10–100% B, 20–21 100% B, 21–23 min 100–10%B, 23–30 min 10% B.

### Flowcytometry

U87MG cells were washed in buffer and stained with either an in-house produced anti-uPAR antibody (3μg/ml)[24], an irrelevant IgG isotype matched control antibody (3μg/ml; 14–4714 eBioscience, San Diego, CA, USA) or blank buffer control for 1 h in 4°C on a shaking table. The cells were washed 3 times with buffer (PBS containing 0.5% HSA and 0.1% natriumazid) before staining with a secondary fluorescent detection antibody (goat-anti-mouse-PE 1/500; Santa Cruz Biotechnology, Heidelberg, Germany) for 30 min in 4°C on a shaking table. The results were analysed on a BD FACSCanto™.

### Dynamic imaging of ICG-Glu-Glu-AE105

Mice (n = 3) were injected intravenously with 10 nmol ICG-Glu-Glu-AE105 and subjected to dynamic imaging at 1, 2, 4, 8, 12, 24, 48, and 72 h. Before scanning, the mice were anaesthetized with 2% isoflurane and positioned in the prone position. For imaging IVIS Lumina XR and acquisition software Living Image (Caliper life Sciences, Hopkinton, CA, USA) were used. The excitation filter was set to 710 nm and the emission filter was set in the ICG. Acquisition was recorded in auto-setting to achieve the best acquisition possible.

### Optical imaging with ICG-Glu-Glu-AE105 and ICG

10 nmol of ICG-Glu-Glu-AE105 or ICG were administered to 5 mice each via tail vein injections. 15 h post injection the mice were anaesthetized with 2% isoflurane a full-body optical scan recorded using the acquisition mode outlined above.

After imaging with IVIS Lumina XR the anesthetized mice were moved to a surgical table and real-time tumor imaging with a Fluobeam®800 NIR-camera was performed (Fluoptics, Grenoble, France). The camera was connected to a standard laptop to allow imaging, adjustments of camera functions and recording of images and video sequences. Fluobeam®800 works with a 800 nm excitation laser optimised for ICG imaging.

The TBR values were calculated as tumor-signal divided by background-signal by drawing a ROI over each tumor and place the background ROI in an area with constant background signal. Images from IVIS Lumina XR were processed in the software Living Image, while the images from Fluobeam®800 was processed in ImageJ.

### *In vivo* blocking of ICG-Glu-Glu-AE105 with uPA

A group of mice (n = 4) were randomized into two groups and injected with either 10 nmol ICG-Glu-Glu-AE105 alone or in the presence of 6.7 nmol uPA. The mice were subsequently subjected to dynamic scanning at 1, 2, 4, 6, 8, 10, 12 and 15 h in the Lumina XR and the Fluobeam as described above.

### ELISA

Tumours were homogenized with the Precellys (Bertin technologies, Montigny le Bretonneux, France) and a suspensions containing the tumor lysate were stored at -80°C. The plate was coated with an anti uPAR antibody R2 (3μg/ml)[24] overnight at 4°C. After incubation, 2% BSA was added for 5 min and the plate was washed with buffer. uPAR standard (10 ng/ml) or tumor lysate (diluted 1:20) was added and incubated for 2 h in RT and washed with buffer. A primary antibody (rabbit-anti-uPAR, 1μg/ml) was added to the well and incubated for 30 min in RT and washed. A secondary HRP conjugated anti-rabbit antibody was added (diluted 1:2.500) and incubated for 30 min in RT and washed. The bound HRP conjugated antibody was quantified by adding the substrate (4 OPD tablets in 12 ml, Dako) and left to react until a proper level of color development was achieved. An ELISA reader was used to analyze the plate at 490 nm and 650 nm as reference.

### Fluorescent imaging and uPAR immunohistochemistry

Tissue was formalin fixed in 4% formaldehyde in 24 h and then embedded in paraffin. For all stainings a 4 μm slice tissue were used. Before any staining the sliced tissue were imaged with an Odyssey scanner (LI-COR Biosciences, Lincoln, Nebraska) at 800 nm.

The tissue was incubated for one hour at 60° before being deparaffinized. Thereafter, tissue was pre-treated with proteinase K (Dako, Glostrup, Denmark) for 6 min. The staining was performed with the Dako ARK kit, with the antibody R2 [24] against uPAR.

### Statistical analysis

All data were analysed using SAS software (SAS Institute Inc., version 6.1, NC, USA). Comparison of groups of quantitative variables was done by the student t-test. Comparison of groups with longitudinal observations at different time points combining quantitative and qualitative variables was performed in a mixed model. A group size of n = 10 was used to allow for detection of a difference in TBR of 30% with a power of 80%. A value of p < 0.05 was considered statistically significant.

## Results

Photophysical properties of ICG-Glu-Glu-AE105 was compared to ICG. The absorption spectrum of ICG-AE105 was slightly broadened as compared to the absorption spectrum of ICG, while the fluorescence and excitation spectra were identical for ICG and ICG-AE105 (Fig 1C). Additionally the binding of ICG-Glu-Glu-AE105 to purified uPAR was measured and yielded an IC_50_ ≈ 134 nM compared to 20 nM for the unmodified AE105 [21] (Fig 1B)

Each plasma sample was analyzed by HPLC and the absorbance area at 780 nm was calculated. This value was compared to the standard sample and the plasma sample at 0 min to calculated the percentage of intact ICG-AE105 recovered in the supernatant (see Table 1).

**Table 1:** Plasma stability.

		Intact ICG-AE105 recovered from supernatant (%)	
Sample	HPLC absorbance area (mAU)*	Compared to standard	Compared to plasma sample at 0 min
**Standard**	**94,8026**	** **	** **
**0 min**	**92,1336**	**97,2**	** **
**1 min**	**87,533**	**92,3**	**95**
**5 min**	**82,2329**	**86,7**	**89,3**
**30 min**	**77,4566**	**81,7**	**84,1**

* determined at 780 nm

**Fig 2:**
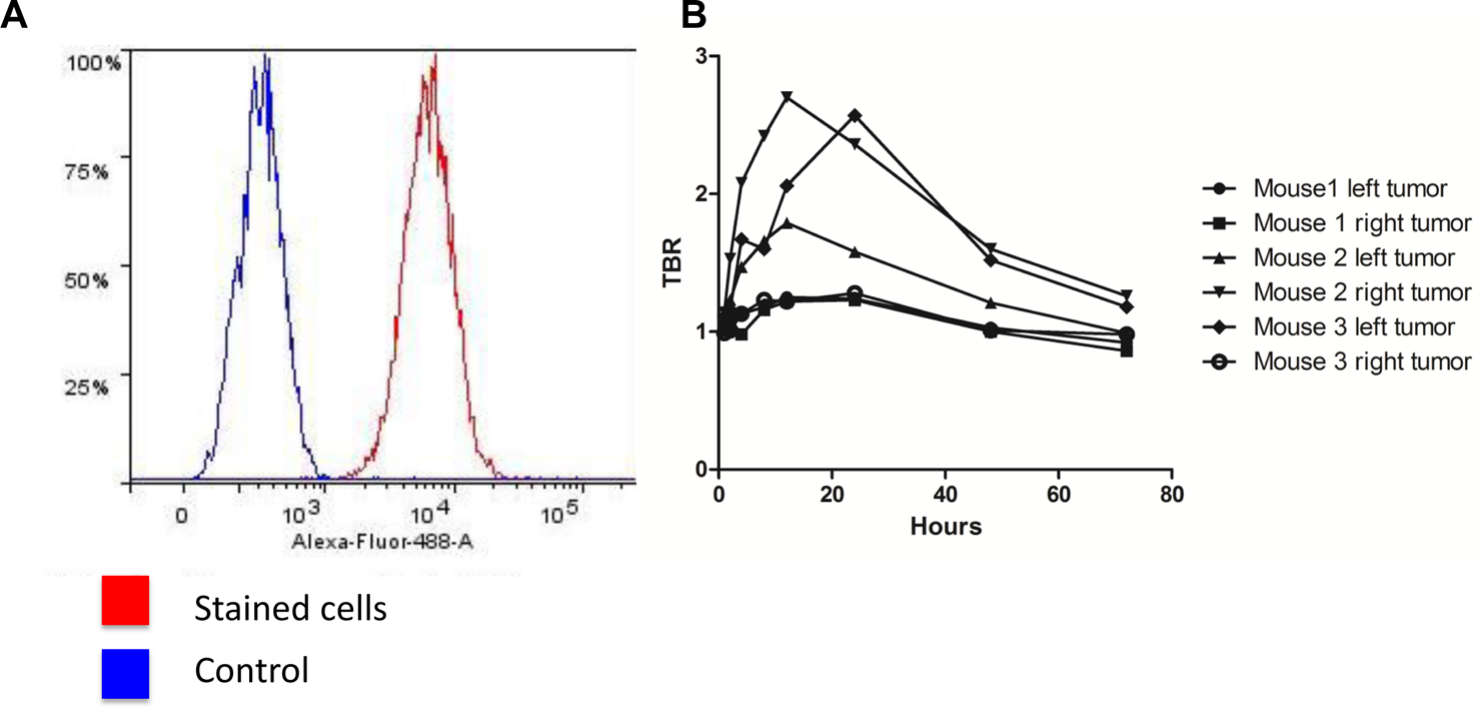
*In vitro* expression of uPAR in the cell line U87MG and *in vivo* TBR values over time (1–72 h). (A) *In vitro* flowcytometry confirms the presence of extracellular uPAR on the U87MG cell line with 98.6% positive for uPAR. (B) Dynamic optical imaging of mice with 10 nmol ICG-Glu-Glu-AE105 at the timepoints 1, 2, 4, 8, 12, 24, 48, 72 h. The graph show clear optimum between 6–24 h. Each graph represents one of two tumors per mouse.

Before any *in vivo* experiments were initiated the expression levels of uPAR on cultured U87MG cells were measured *in vitro* by flowcytometry. The staining with rabbit-anti-uPAR showed a clear right-shift in the fluorescence compared to the control, thus confirming high levels of uPAR expression (Fig 2A). A staining percentage at 98.6% was seen for U87MG.

**Fig 3:**
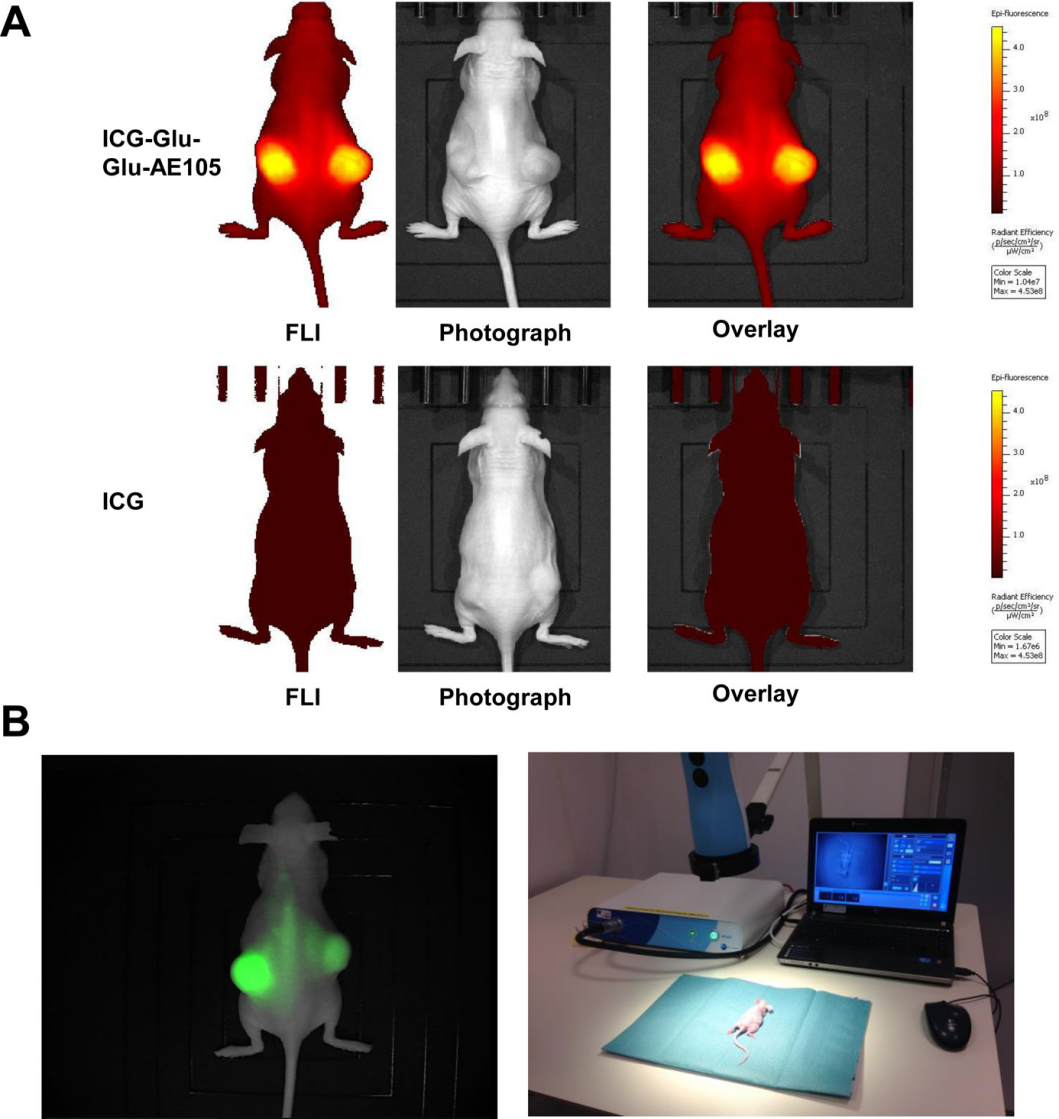
Optical images of tumor bearing mice with ICG-Glu-Glu-AE105 or ICG. (A) Representative optical images of U87MG tumor bearing mice from both groups 15 h post injection (ICG-Glu-Glu-AE105 and ICG) obtained with the IVIS Lumina XR. The images show clear difference in the fluorescent signal from the s.c. tumors with 3.52±0.17 and 1.04±0.04 respectively. The images are shown within the same scalebar to allow for direct comparison. (B) Image from the Fluobeam^®^800 and the Fluobeam setup. A Fluobeam image of a representative mouse with s.c U87MG tumors. The difference in intensity of the two tumors is a result of size of the tumors and the optical properties of the camera. The representative image from the fluobeam camera shows similar signal as the black-box imager IVIS Lumina XR. This underlines the translational potential of ICG-Glu-Glu-AE105.

**Fig 4:**
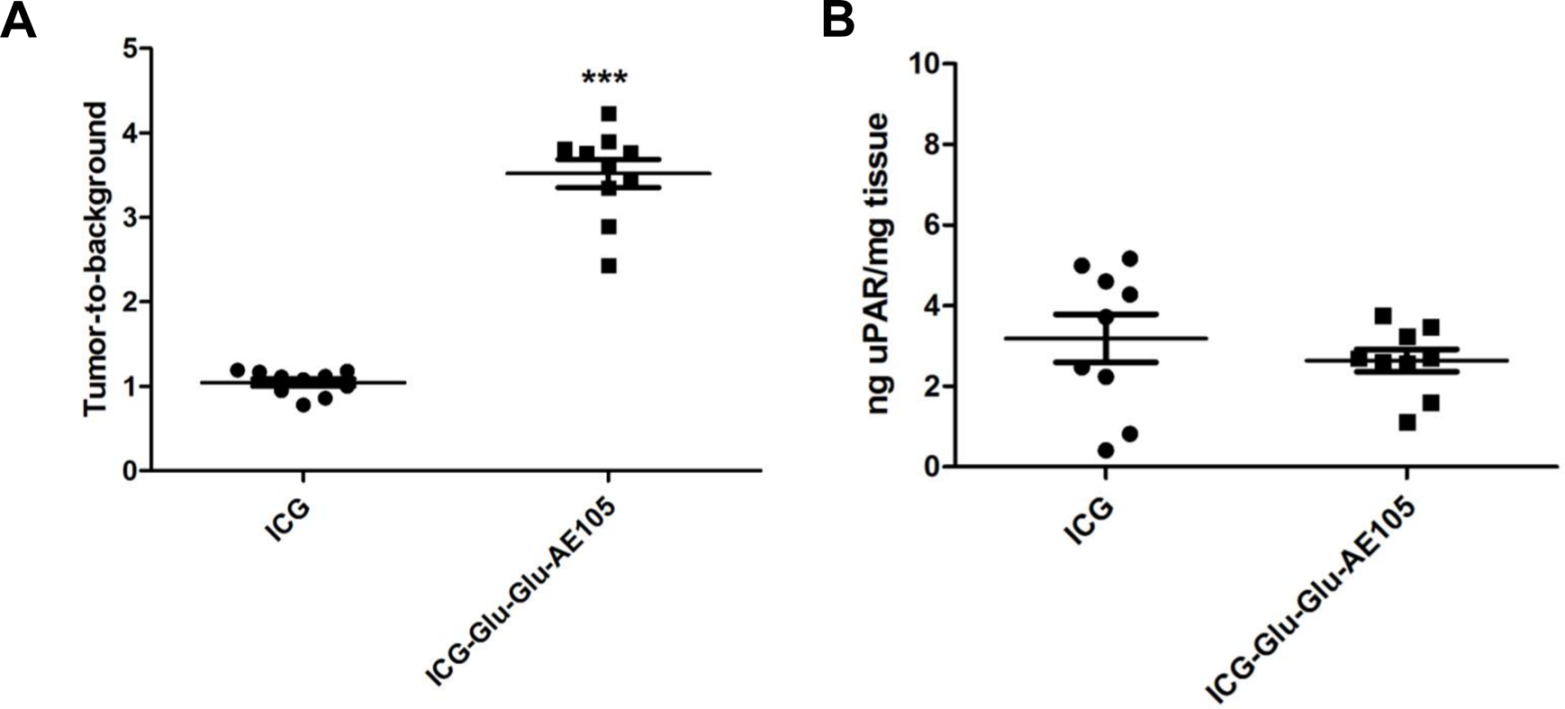
uPAR expression measured by optical signal and ELISA assay. (A) The mean TBR value was significantly different (1.04±0.04 for ICG and 3.52±0.17 for ICG-Glu-Glu-AE105, p<0.0001), while the uPAR expression per mg tissue was almost identical and supports the hypothesis that the difference in ICG-Glu-Glu-AE105 and ICG signal is due to uPAR binding.

A pilot study for identification of the optimal scanning time had shown a time window at 6–24 h post injection to provide the best TBR value (Fig 2B), and 15 hours was chosen for further imaging experiments. To investigate the specificity of the ICG-Glu-GLU-AE105 targeting, a group of mice (n = 5) were scanned 15 h post injection with ICG-Glu-Glu-AE105 in the IVIS Lumina XR. A high uptake in the tumor was observed (Fig 3A) and quantitative analysis of the tumor and background uptake, showed a TBR of 3.52±0.17 (n = 10) (Fig 4A). Next, a group of mice (n = 5) were imaged with ICG alone. No uptake was seen in the tumor at 15 h post injection, and the TBR for ICG was 1.04±0.04 (n = 10) (Fig 4A). When comparing the two groups, a significant difference (p-value < 0.001) between the group receiving ICG-Glu-Glu-AE105 and the group receiving ICG alone, was observed. No adverse effects were observed in any of the mice.

Next, to demonstrate the translational potential of our probe, mice (n = 5) injected with ICG-Glu-Glu-AE105 was also imaged with the clinically approved camera Fluobeam^®^ 800 (Fluoptics) (Fig 3B). Clear tumor identification and delineation from surrounding healthy tissue was possible due to high uptake of ICG-Glu-Glu-AE105 (Fig 3B). In addition surgical removal of tumors guided by real-time fluocescence imaging was possible. This imaging modality gave similar TBR (3.58±0.29) as the IVIS Lumina XR and thus confirms the translational potential of ICG-Glu-Glu-AE105 probe.

All tumors from both groups of mice were resected after the last scan and the uPAR expression in the tumor lysates were analysed. uPAR expression levels were identical in each group with 3.19±0.59 ng uPAR/mg tissue and 2.64±0.28 ng uPAR/mg tissue in the ICG and ICG-Glu-Glu-AE105 group, respectively (Fig 4B), thus confirming that the higher uptake of ICG-Glu-Glu-AE105 compared to ICG could not be explained by higher uPAR levels.

**Fig 5:**
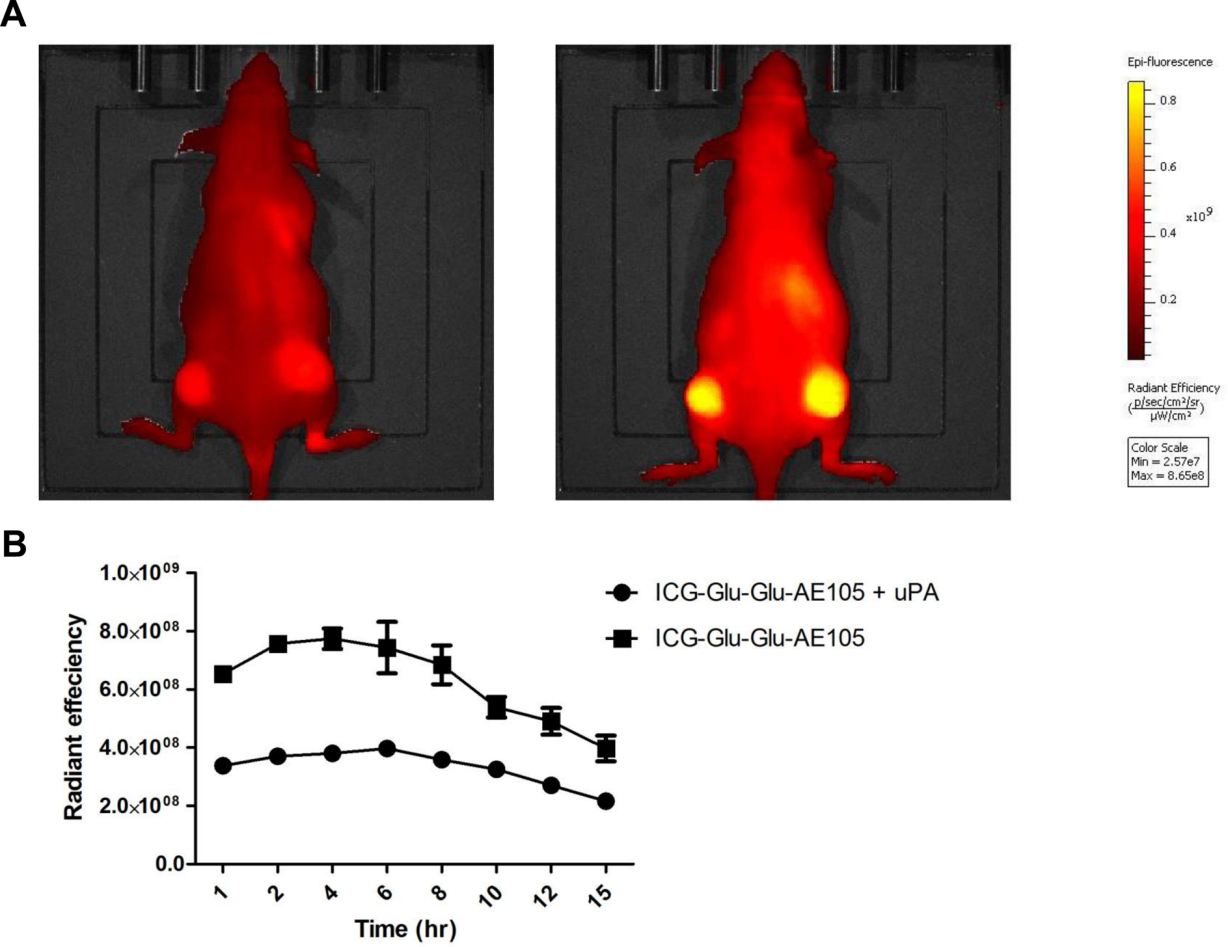
*In vivo* blocking of ICG-Glu-Glu-AE105 by uPA, the natural ligand. (A) Representative images obtained by the IVIS Lumina XR at 710 nm showing a mouse receiving uPA simultaneously with ICG-Glu-Glu-AE105 resulting in decreased signal compared to a mouse only receiving ICG-Glu-Glu-AE105. (B) Two groups of mice (n = 4) were dynamically scanned with either ICG-Glu-Glu-AE105 + uPA or ICG-Glu-Glu-AE105. In all timepoints the two groups were significantly different with the group receiving only ICG-Glu-Glu-E105 having a 2 fold higher signal.

To further investigate the specificity, mice (n = 4) were next injected with either 10 nmol of ICG-Glu-Glu-AE105 alone or together with 6.7 nmol uPA (Fig 5A). Saturating uPAR with its natural protease ligand uPA resulted in a decrease in the fluorescence signal from the xenotransplanted tumors. Fig 5B shows the fluorescence signal over time and a mean decrease of the max fluorescence signal of 46.78%±3.65% was observed in the group receiving ICG-Glu-Glu-AE105 + uPA compared to the group only receiving ICG-Glu-Glu-AE105. Comparing the two groups in a mixed model the blocking was highly significant (p < 0.0001) and a significant difference was present during all time points investigated.

**Fig 6:**
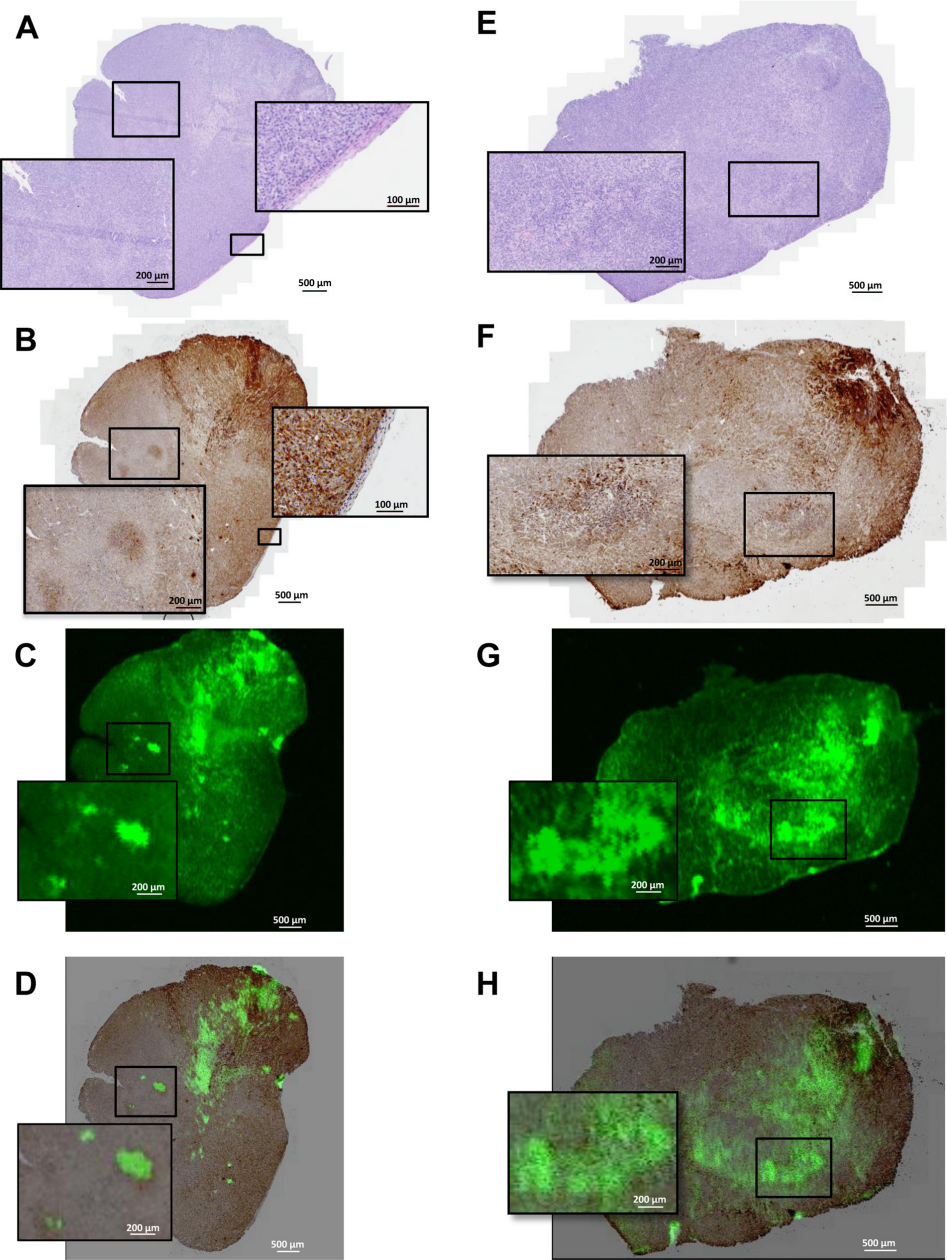
*Ex Vivo* histology and fluorescence images of s.c. U87MG tumor tissue sections. Shown here is from the top H&E staining (A,E), uPAR staining by immunohistochemistry (B,F), uPAR staining by fluorescence from the injected ICG-Glu-Glu-AE105 (C,G) and merged uPAR IHC and fluorescence imaging (E,H). In the first row panel A shows the H&E staining of the tumor. uPAR IHC staining (B) illustrate two clear islands of uPAR positive cells which are also depicted with fluorescence imaging (C). Co-localization of uPAR expression and ICG-Glu-Glu-AE105 fluorescence is shown I the merged IHC and fluorescence image (D). Additionally in panel B the border between human xenograft tissue and mouse stroma is seen. In the second row another tumor speciment is shown wht H&E staining in panel E. The uPAR staining (F) show heterogeneous uPAR expression and the enlarged image show an island with cells expressing higher amount of uPAR. This is also depicted in panel G where the same island of cells can be located by fluorescence. The merged uPAR IHC and fluorescence image (H) show the co-localization of uPAR expression and fluorescent signal.

Finally, tumor tissue sections were then imaged for fluorescent distribution and showed a heterogeneous accumulation of ICG-Glu-Glu-AE105 in the sections (Fig 6C and 6G). Compared to the uPAR immuhistochemistry staining of the same sections (Fig 6B and 6F), co-localization of the uPAR staining and the fluorescent probe signal was observed (Fig 6D and 6H), to further confirm the specificity of ICG-Glu-GLU-AE105 towards human uPAR.

## Discussion

In the present study, a new optical imaging uPAR-targeted probe ICG-Glu-Glu-AE105 was characterized *in vitro* and *in vivo* in a human glioblastoma xenograft mouse model. Our results described in the present study were encouraging as proof-of-concept and prompt further studies and clinical translation of the probe.

In our study the novel optical probes was investigated for its photophysical properties, binding affinity and plasma stability and was found to mimic both the peptide AE105 and the fluorophors properties, and as conjugated probe exhibit high stability in plasma. Further the study showed a specific *in vivo* uptake of ICG-Glu-Glu-AE105 in the human glioblastoma xenograft model. It was possible to obtain a maximal TBR value in a time window span of 6–24 h post injection irrespective of the imaging platform, a preclinical (IVIS Lumina XR) or clinical camera (Fluobeam^®^800). Furthermore *in vivo* imaging with non-targeted ICG showed no uptake in tumor 15 h post injection. A significant reduction of 47%±4% in the signal was obtained when a blocking step with the natural ligand for uPAR uPA was included in the imaging protocol. Microanatomical co-registration of ICG-Glu-Glu-AE105 fluorescence activity and uPAR staining on histological slides demonstrated further the uPAR specific uptake of the optical probe in tumor tissue. Taken together ICG-Glu-glu-AE105 may therefore be considered a specific uPAR targeting optical probe.

The prognosis after surgery is highly dependent on the margin status. Several studies have shown increased recurrence-rates when positive margins were present after surgery [8], and positive margins after resection is a major problem in several types of solid cancer, e.g. 38% in breast cancer [1]. Therefore an intra-operative imaging modality is needed to visualize the positive tumor margins during surgery.

The field of intra-operative optical imaging has developed rapidly the past decade partly due to a pronounced unmet clinical need for accurate real-time imaging during cancer resection and partly due to the fast development of clinically available optical camera systems [25]. This has pushed the need for targeted optical probes identifying the diseased tissue. The first clinical trial with a targeted fluorescent probe against the folate receptor has already been conducted in ovarian cancer [26], and there is a great need for more specific probes suitable for different cancer types.

Many designs of optical probes have been tried. Several groups have investigated probes targeting the EGFR receptor [27–29], integrin α_v_β_3_ [30,31] and HER1 and HER2 [27,32]. The overexpression of uPAR in multiple cancers and the high expression in the border of the tumor and in the surrounding stroma makes uPAR an attractive target for intraoperative optical imaging. Targeting uPAR has already been performed using other imaging modalities [33] such as PET [16–19], SPECT [34], MR [35] and optical imaging [36–38]. Yang and colleagues were the first to develop a uPAR targeted optical probe [38]. Their study investigated several combinations of targeting vector and fluorophor including the amino terminal fragment (ATF) of the binding domain of uPA (mouse or human), magnetic iron oxide nanoparticles, NIR-830, Cy5.5 and IRDye800CW. In summary these optical probes showed promising results in tumor delineation in an orthotopic breast cancer model and high TBR values. Unfortunately, the time course for tumor imaging was not optimal with maximum TBR at 72 h post injection and a persistent signal throughout 2 weeks. Furthermore, ATF and nanoparticles have not been tested for clinical use and a clinical translation in the near future is not likely. Optical imaging of uPAR with antibody based probes have also been investigated and shown promising results [37,39,40]. But except for one study where the fluorophor was conjugated to antibodies most were not relevant for clinical translation. Boonstra et al. used an antibody conjugated with a clinically relevant fluorophor ZW800-1 which has shown outstanding TBR properties comparing to other fluorophors [37]. In contrast to our peptide-based probe, these antibody probes have been used as targeting vectors because of the ease of conjugation, the well-known high affinity for the target and commercial availability. However, using antibodies for *in vivo* imaging has a number of limitations. The size of an antibody influences the pharmacological profile, and results in a long plasma half-life. An early high TBR value is difficult to achieve with a long plasma half-life and an acceptable TBR value is therefore only achievable 1–3 days after injection [27,29,32] thus limiting the clinical usefulness and thereby the translation potential. If smaller peptides, as is our nonapeptide, are used, an optimal imaging time-point can be achieved already 3–6 hours after injection as a result of the faster clearance time [41]. This has already been shown for the peptide AE105 by Sun et al. though with the non-translational fluorophor Cy5.5 [42]. In our study the TBR value reached a steady level already after 6 h and was sustained until 24 h post injection where the signal started to decrease, thus providing a broad clinically useful time-window for imaging.

Besides the peptide, the fluorophor also has influence on the probe property. Numerous fluorophors in the NIR window with different characteristics are available. ICG has its advantage in its long history in clinical use [43]. ICG is approved by FDA and EMA and has a well-established safety profile, thus paving the way for a potential fast clinical translation. However, several studies [38,44] have shown that conjugation of ICG to an antibody decreases the fluorescent signal from ICG. A comparison of ICG and ICG-Glu-Glu-AE105 *in vitro* showed a 2-fold decrease in fluorescence intensity for the conjugated probe. Even though a decrease in fluorescence intensity is seen *in vitro* the probe still comprises enough fluorescence intensity to yield a high TBR. The fluorescent properties of ICG has been surpassed by other upcoming fluorophors such as IRDye 800CW [25]. This newly developed fluorophor exhibits features as higher quantum yield, easier conjugation and hydrophilicity. Especially the hydrophobicity of ICG seems to be an important feature considering the reduction in binding affinity of the probe found in this study due to conjugation to ICG. However, IRDye800 has only recently been tested in a single clinical study [45], paving the way for IRDye800 to be used in translational research.

The stability of ICG-Glu-Glu-AE105 was investigated *in vitro* by measuring the plasma stability on HPLC and *in vivo* by injecting equivalent amount of ICG. The plasma stability results indicate that 92.3%, 86.7% and 81.7% of the probe is intact compared to a standard sample after 1, 5 and 30 min. Imaging of ICG alone showed no uptake 15 h post injection thus indicating that the signal of ICG-Glu-Glu-AE105 in Fig 3 was due to uPAR targeting and not instability or degradation of the probe. For specificity study a bolus of uPA together with ICG-Glu-Glu-AE105 was injected. Blocking the binding of ICG-Glu-Glu-AE105 with the natural ligand uPA by almost 50% underlines that the optical signal observed in the xenograft tumors was emitted by ICG-Glu-Glu-AE105 due to targeting of the uPAR receptor in the tissue. In addition, paraffin embedded tumor sections were imaged *ex vivo* by the Odyssey scanner and immunostained for uPAR. This showed point-to-point co-localization of uPAR expression and ICG-Glu-Glu-AE105-binding.

To investigate if ICG-Glu-Glu-AE105 has potential to delineate tumors during surgery, studies where orthotopical xenograft tumor models are used for fluorescent guided surgery and recurrence after resection is needed and will also assess the minimum size of tumor deposit necessary for a fluorescent signal.

Our uPAR targeted probe consists of two molecules already used clinically. AE105 has recently been used in first-in-human phase I study as PET ligand by our group [46] and ICG has been used for optical guidance in lymphatic drainage in numerous studies [47]. This should therefore make clinical translation of our novel uPAR-targeting probe more plausible.

We conclude that the aim to develop a targeted ICG probe, exhibiting high affinity and specificity towards uPAR and high *in vivo* stability was obtained. Results from our study show that the probe ICG-Glu-Glu-AE105 possesses all these properties. The findings highlight the potential for clinical translation of this NIR optical probe for intraoperative optical-guided resection of uPAR positive cancer lesions.
